# Supplementary material for: Bacterial Adaptation to Venom in Snakes and Arachnida
Source: Microbiol Spectr. 2022 May 23;10(3):e02408-21. doi: 10.1128/spectrum.02408-21 (PMC9248900; doi:10.1128/spectrum.02408-21)
Supplement: SUPPLEMENTAL FILE 1 — Supplemental material. Download spectrum.02408-21-s001.pdf, PDF file, 2.4 MB [file spectrum.02408-21-s001.pdf]

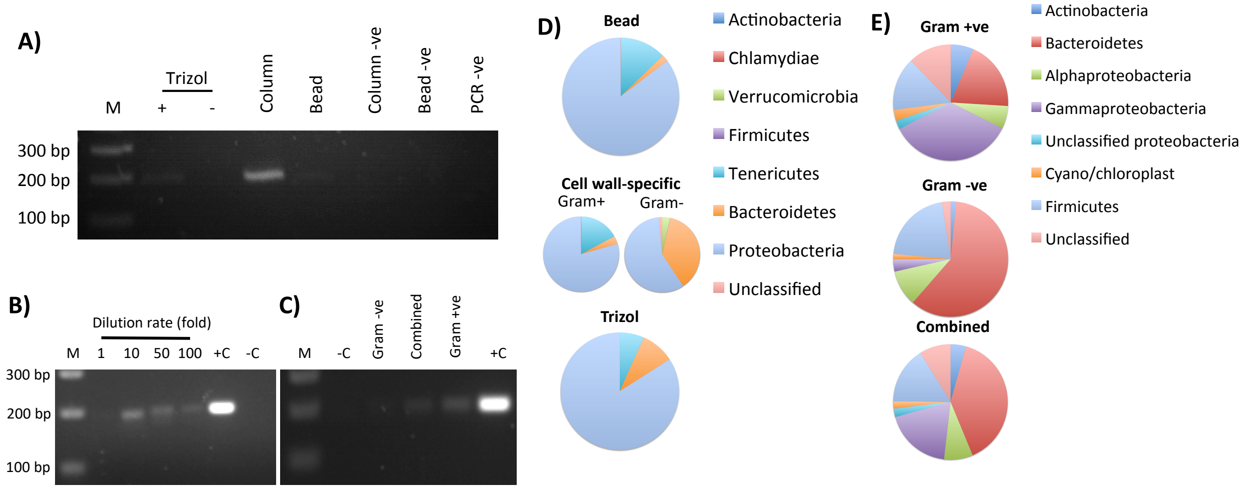

Figure S1: 16S Ribosomal RNA gene PCR output and phylogenetic differences on account of venom collection and extraction methods. The choice of extraction method (phenol-chloroform-based: Trizol; column based; magnetic bead based) impacts significantly on the recovery and amplification of bacterial DNA from lyophilized *B. atrox* venom (A). This bacterial DNA is not an artefact of lyophilisation process contamination as detection is maintained in aseptically collected, flash-frozen *Bitis arietans* venom, nor is it an artefact of diluent contamination by 18 MΩ water confirmed 16S free by PCR; however, >10x dilution of venom is necessary for PCR to progress (B). The yield of bacterial DNA is a function of upstream cell lysis methods selectivity for Gram +ve or Gram –ve cell walls (C). The cell lysis and extraction methodology also directly impact upon microbial diversity profiles as determined by 16S rRNA phylogenetics for either lyophilised (D) or aseptically collected, flash-frozen venoms (E), , with combined use of cell wall-specific extraction methods yielding more balanced profiles.. +C: positive control; -C: negative control; -ve: method specific negative controls.

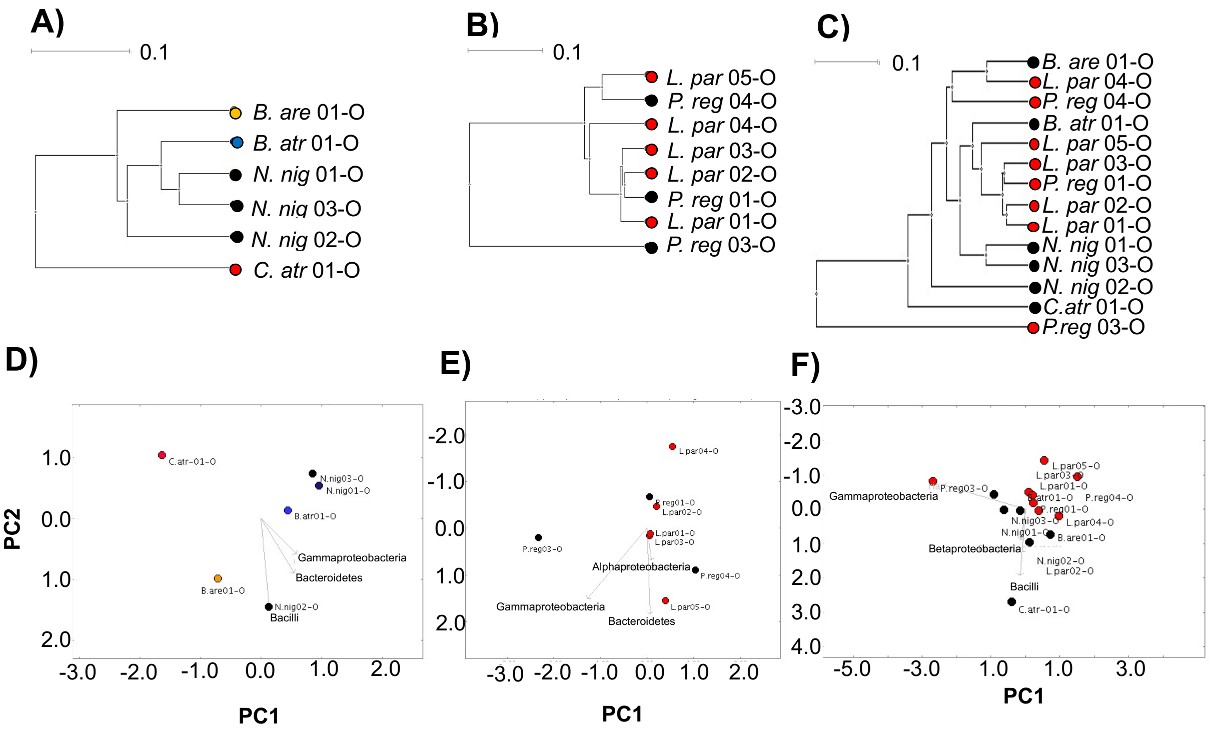

Figure S2: Comparison of the oral microbiomes of snakes and spiders suggests their oral microbiota is not host-species specific. UPGMA tree (A - C) and PCoA (D – F) analysis (Bray-Curtis indeces) of the oral microbiome diversity of snakes (A, D; individual species identified by independently coloured dots), spiders (B, E; *L. parahybana*: red dots, *P. regalis*: black dots), or vertebrate vs invertebrate animals (C, F; black vs red dots), as determined by 16S rRNA phylogenetic analysis at class level indicate no host species-specific relationships. Dots represent single captivity individuals, labelled with short species name, enumerated for individual number and identified for the oral/fang (O) nature of the sample.

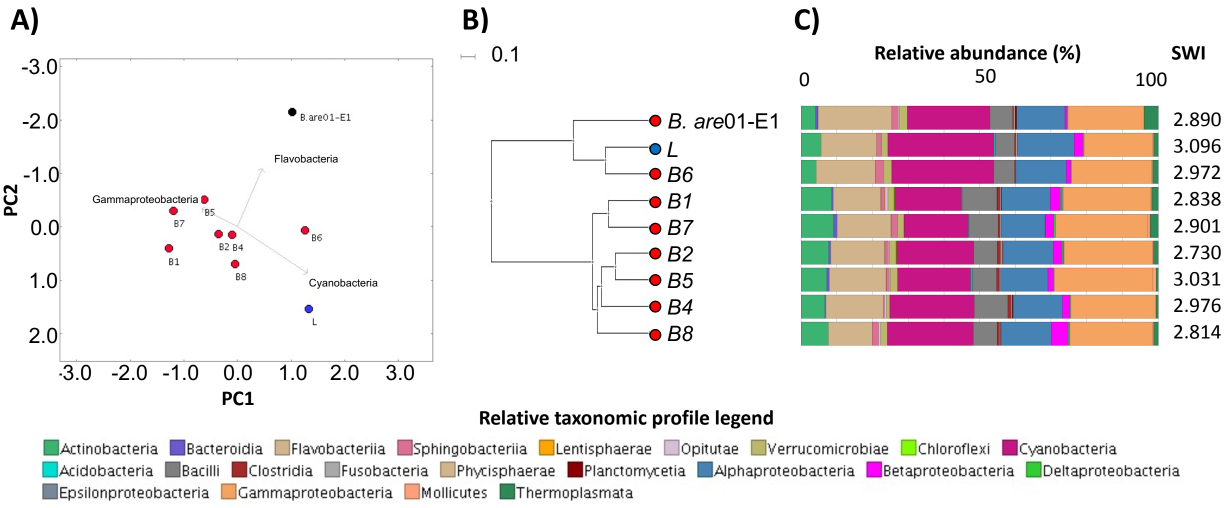

Figure S3: The origin of a *B. arietans* snake does not appear to influence the microbiome profile in the venom of each animal. *B. arietans* venom microbiome profiles do not present substantial differences on account of host geographical origin as determined by A) PCoA, B) UPGMA tree and C) class-level taxonomic profiling following 16S rRNA phylogenetic analysis. Dots in (A) and (B) represent individual animal data, are coloured and labelled by animal origin and number (red B1-B8: wild; blue L: lyophilised captivity (Latoxan); black B. are01-E1: flash-frozen captivity, Venomtech). Relative taxonomic diversity profiles in (C) are aligned to the UPGMA tree sample labels, with the Shannon-Wiener Index (SWI) of each sample indicated. Sample B3 was removed from the analysis due to the ~100x lower read depth yield from this sample compared to all other *B. arietans* samples.

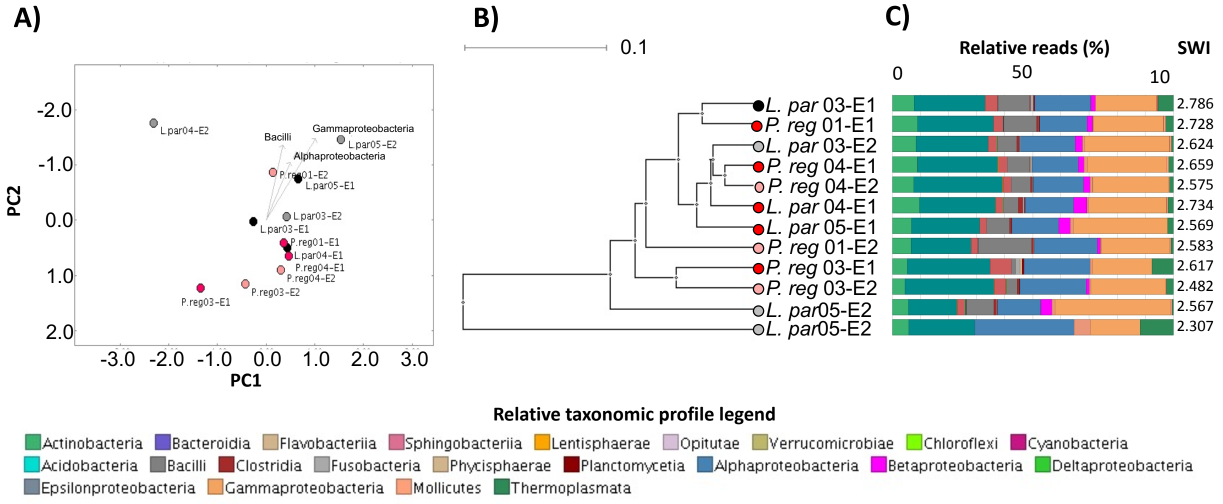

Figure S4: Spider venom microbiome profiles suggest closer relationships between consecutive envenomation samples within *P. regalis* individuals. Spider venom microbiomes were compared by A) PCoA, B) UPGMA tree and C) class-level taxonomic profiling following 16S rRNA phylogenetic analysis. Dots in (A) and (B) represent individual animal data, are colored/labelled by species and envenomation number (black and grey: *L. parahybana* envenomation 1 (E1) and 2 (E2) respectively; red and pink: *P. regalis* E1 and E2 respectively). Relative taxonomic diversity profiles in (C) are aligned to the UPGMA tree sample labels, with the Shannon-Weiner Index (SWI) of each sample indicated.

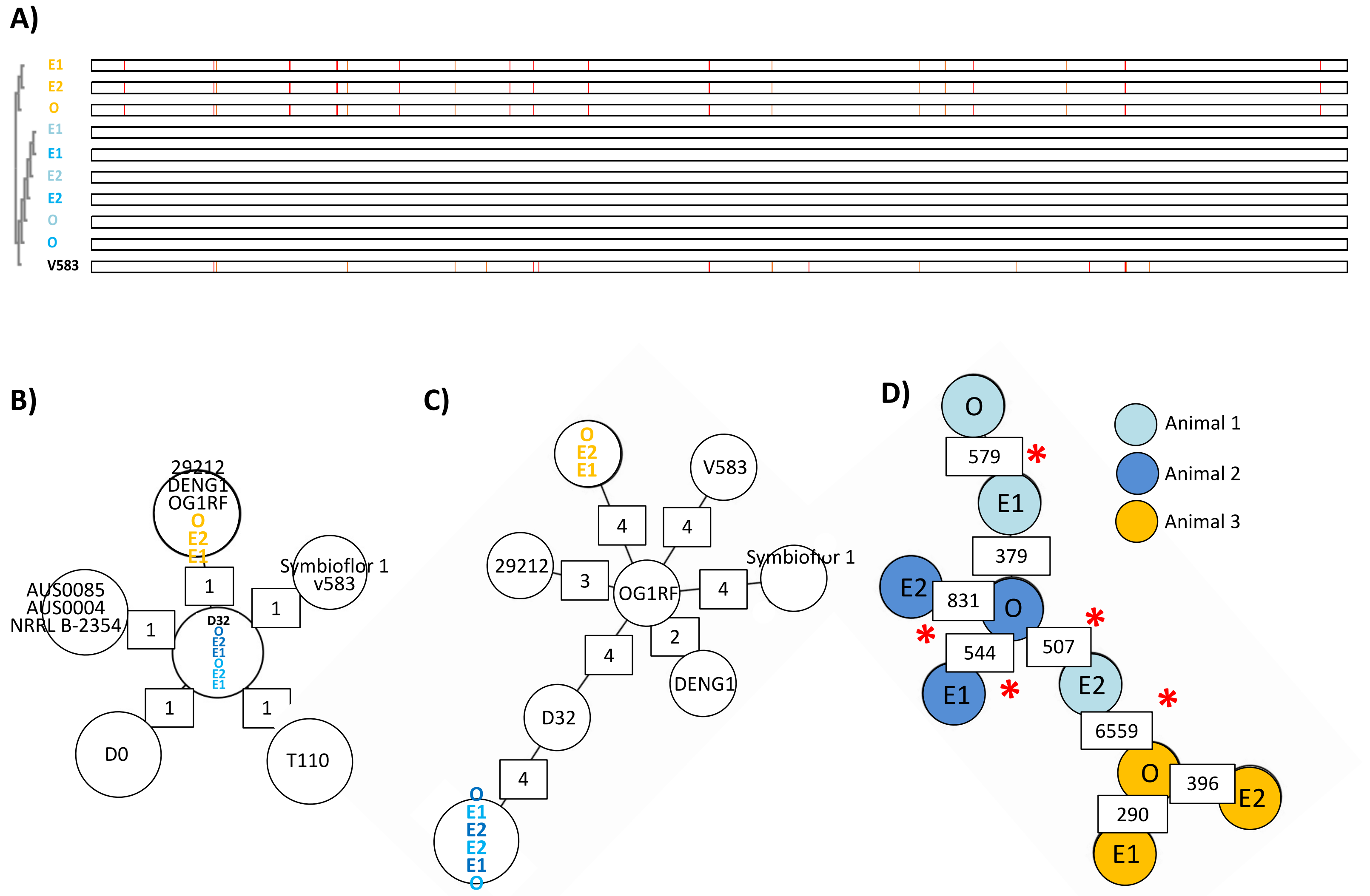
A)
E1
E2
O
E1
E1
E2
E2
O
O
V583
B)
29212
DENG1
OG1RF
O
E2
E1
AUS0085
AUS0004
NRRL B-2354
Symbioflor 1
v583
D32
O
E2
E1
O
E2
E1
D0
T110
1
1
1
1
1
C)
O
E2
E1
V583
29212
OG1RF
Symbioflor 1
DENG1
D32
O
E1
E2
E2
E1
O
4
4
3
4
2
4
4
D)
O
579
E1
379
E2
831
O
544
507
E1
E2
6559
O
396
E2
290
E1
Animal 1
Animal 2
Animal 3

Fig. S5: MSA, MST and cgMLST define two novel *E. faecalis* sequence types isolated across *N. nigricollis* venom and oral cavities. Blinded MSA (A) of the *KatA* gene sequence across the nine *E. faecalis isolates* obtained from *N. nigricollis* oral swabs (O) and two consecutive envenomation samples (E1 and E2) from three independent animals (light blue (N.nig01), dark blue (N.nig02) and orange (N.nig03)) defines two alleles distinct to the V583 reference sequence (bottom lane). Base conservation is defined by similarity to the animal 1 and 2 *KatA* sequence using BoxShade v.3.3.1 on mobyle.pasteur.fr. Each pixel column represents a different nucleotide with orange and red columns indicating increasingly different nucleotides. Blinded MST analysis of these nine isolates against B) *E. faecalis* and *E. faecium* reference genomes (distance calculations based on *E. faecium* MLST), C) *E. faecalis* reference genomes with partial incidence locus data removed, and D) a custom cgMLST schema derived from *E. faecalis* OG1RF, D32 and DENG1 including loci with partial data between all study isolates (8101 targets). Allelic differences in excess of 5% of the cgMLST schema are highlighted by '*'. Reference genomes: *E. faecalis*: V583, OG1RF, D32, DENG1, 29212, Symbioflor 1; *E. faecium*: T110; AUS0085, Aus0004, NRRL B-2354, DO.

```
A)>pstS_E.faecalis_N.nig03_venom
GTGACCATTGGAAATTCCGATGTGTTTGCCGAAGAGAAAGATGGCGTGGATGCCTCTAAACTAGTTGATCATCGGGTGGCCGTGGTTGGTATGGGACCAGTGG
TCAACAAAGAAGTCGGCGTGAAGAATTTAACAAAGCAACAATTGATTGATGTCTTTACTGGCAAAGTCAAAAACTGGAAAGAAGTGGGCGGCAAAGATCAAGA
AATCGTCGTAATAAACCGCGCAAACGGAAGTGGCACCCGAGCAACATTTGAAAAATGGGGCTTAGATGGAGCTAAACCAGTTCAATCACAAGAACAAGATTCT
TCGGGAACAGTTCGTAAAATTGTTGAACAAACACCAGGAGCAATCAGCTATTTAGCTTTTTCTTATATGGATGATTCCACCGTTGCTTTAAGCATTGATGGTG
TTGAACCAAAAGAAGAACACGTGAAAGACAATTCATGGAAAATCTGGTCTTATGAACATATGTATACAAAAGGGGAGCCTAATAAAGAAGTAAAAGCCTTCTT
AGACTATATGGTCACTGATGATGTTCAAAAAACAATTGTCAAAGACTTAGGTTATTTAGCCATCACAG

B)>yqiL_E.faecalis_N.nig03_venom
ATGTTACAACACAACTTTTAAAAAGACATTCCACTATTTCTGAAGAAATTGATCAAGTAATCTTTGGAAATGTTTTACAAGCTGGAAATGGGCAAAATCCCGC
ACGACAAATAGCAATAAACAGCGGTTTGTCTCATGAAATTCCCGCCATGACGGTTAATGAGGTCTGCGGATCAGGCATGAAGGCCGTTATTTTGGCGAAACAA
TTGATTCAATTAGGAGAAGCGGAAGTTTTAATTGCTGGTGGGATTGAGAATATGTCCCAAGCACCTAAATTACAACGATTTAATTACGAAACAGAAAGCTACG
ATGCGCCTTTTTCTAGTATGATGTATGATGGGTTAACGGATGCCTTTAGTGGTCAGGCAATGGGCTTAACTGCTGAAAATGTGGCCGAAAGTATCATGTAAC
TAGAGAAGAGCAAGATCAATTTTC

C)yqiL_E.faecalis_N.nig02_E1      AAAGCTACGATGCGCCTT-TTTCTAGTATGATGTATGATGGGTTAACGGATGCCTTTAGT
  yqiL_E.faecalis_allele-8        AAAGCTACGATGCGCCTT-TTTCTAGTATGATGTATGATGGGTTAACGGATGCCTTTAGT
  yqiL_E.faecalis_N.nig02_E2      AAAGCTACGATGCGCCTTTTTTCTAGTATGATGTATGATGGGTTAACGGATGCCTTTAGT
                                  ****************** *****************************************

  yqiL_E.faecalis_N.nig02_E1      GGTCAGGCAATG-GCTTAACTGCTGAAAATGTGGCCGAAAGTATCATGTAACTAGAGAA
  yqiL_E.faecalis_allele-8        GGTCAGGCAATGGGCTTAACTGCTGAAAATGTGGCCGAAAGTATCATGTAACTAGAGAA
  yqiL_E.faecalis_N.nig02_E2      GGTCAGGCAATGGGCTTAACTGCTGAAAATGTGGCCGAAAGTATCATGTAACTAGAGAA
                                  ************ **********************************************
```

Figure S6: Novel *pstS* and *yqiL* allele sequences obtained from *N. nigricollis* venom-derived *E. faecalis.* The sequences of the novel *pstS* (A) and *yqiL* (B) alleles found in a novel *E. faecalis* sequence type obtained from *N. nigricollis* venom (animal 3). Clustal omega alignments of the *yqiL* sequences (C) from *E. faecalis* isolates derived from animal 2 venom against *E. faecalis* *yqiL* allele 8 found in the orally-derived isolate. The alignment is focused to positions 301-319 of the 436 nt allele and single base pair indels are highlighted in red.

A)

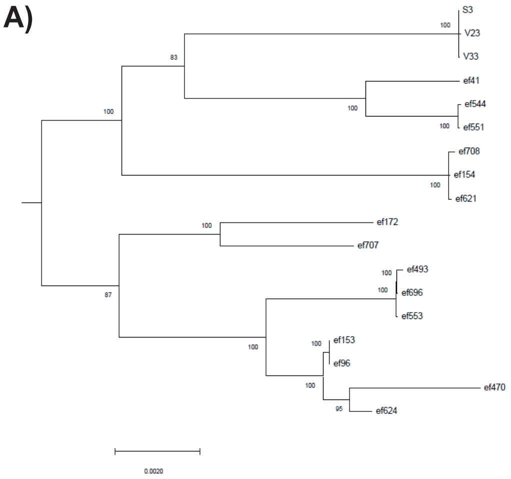

Enterococcus faecalis S3 - *N. nigricollis animal 3 - O*
Enterococcus faecalis V23 - *N. nigricollis animal 3 - E2*
Enterococcus faecalis V33 - *N. nigricollis animal 3 - E1*
Enterococcus faecalis Fly1 - Fruit fly
Enterococcus faecalis CVM N59592F - Turkey
Enterococcus faecalis CVM N60280F - Pig
Enterococcus faecalis G881 - Urine
Enterococcus faecalis RMC5 - Clinical
Enterococcus faecalis CVM N53761 - Chicken Wings
Enterococcus faecalis TR197 - Blood
Enterococcus faecalis G882 - Urine
Enterococcus faecalis S15 - River water
Enterococcus faecalis 91EA1 - Chicken breast
Enterococcus faecalis CVM N59959F - Chicken
Enterococcus faecalis D3 - Pig
Enterococcus faecalis 7330245-2 - Pig Faeces
Enterococcus faecalis P. En090 - Pig Faeces *i* CVM N53457 - Pork Chop

B)

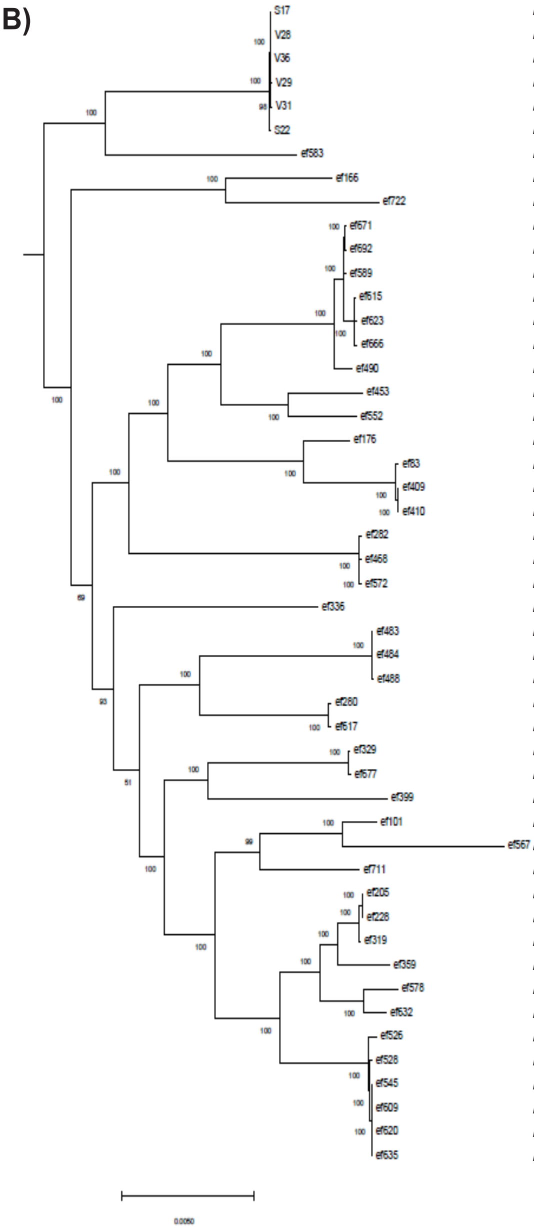

Enterococcus faecalis S17 - *N. nigricollis animal 2 - O*
Enterococcus faecalis V28 - *N. nigricollis animal 2 - E2*
Enterococcus faecalis V36 - *N. nigricollis animal 1 - E1*
Enterococcus faecalis V29 - *N. nigricollis animal 1 - E2*
Enterococcus faecalis V31 - *N. nigricollis animal 2 - E1*
Enterococcus faecalis S22 - *N. nigricollis animal 1 - O*
Enterococcus faecalis CVM N59457F
Enterococcus faecalis 39-5 - Oral isolate (periodontitis)
Enterococcus faecalis NCTC8176 - danish blue cheese
Enterococcus faecalis 234EA1 - Chicken breast
Enterococcus faecalis 61EA1 - Chicken breast
Enterococcus faecalis CVM N59606F - Pig
Enterococcus faecalis CVM N54576 - Ground turkey
Enterococcus faecalis CVM N53661 - Chicken breast
Enterococcus faecalis 16EA1 - Chicken breast
Enterococcus faecalis S13 - Chicken feces
Enterococcus faecalis L9 - Pig Rectal swab
Enterococcus faecalis CVM N59963F - Heifer
Enterococcus faecalis ATCC 6055 - Milk
Enterococcus faecalis B16457 - Human
Enterococcus faecalis 566.rep1_EFLS - Human
Enterococcus faecalis 566.rep2_EFLS - Human
Enterococcus faecalis B83616-1
Enterococcus faecalis Enfs94 - Human (stool)
Enterococcus faecalis CVM N59591F - Chicken
Enterococcus faecalis FL2 - Freshwater
Enterococcus faecalis WZ21-1 - Fermented goat milk
Enterococcus faecalis WZ34-2 - Fermented goat milk
Enterococcus faecalis YM39-2 - Fermented goat milk
Enterococcus faecalis 02-MB-BW-10
Enterococcus faecalis CVM N54553 - Ground turkey
Enterococcus faecalis EnGen0424 - Human
Enterococcus faecalis 277EA1 - Chicken breast
Enterococcus faecalis 222_EFLS - Human
Enterococcus faecalis 7430315-3 - Pig Faeces
Enterococcus faecalis CVM N59862F - Pig
Enterococcus faecalis G883
Enterococcus faecalis Com 2 - Human stool (healthy volunteer)
Enterococcus faecalis Com1 - Human stool (healthy volunteer)
Enterococcus faecalis EnGen0414 - Human
Enterococcus faecalis 17 - Pig
Enterococcus faecalis CVM N59508F - Pig
Enterococcus faecalis CVM N52729 - Pork Chop
Enterococcus faecalis UBA5708 - Environment (Terrestrial) metagenome
Enterococcus faecalis UBA5875 - Environment (Terrestrial) metagenome
Enterococcus faecalis CVM N59600F - Chicken
Enterococcus faecalis CVM N55119 - Chicken Wings
Enterococcus faecalis CVM N54118 - Ground turkey
Enterococcus faecalis CVM N54121 - Ground turkey

Figure S7: Source of *E. faecalis* strains with genomes closely related to venom-tolerant strains isolated from *N. nigricollis* venom. The genome record metadata available for the closest *E. faecalis* isolates related to (A) group A and (B) group B *N. nigricollis* venom isolates (subtrees extracted from the original 734 *E. faecalis* strain core genome tree) are depicted.

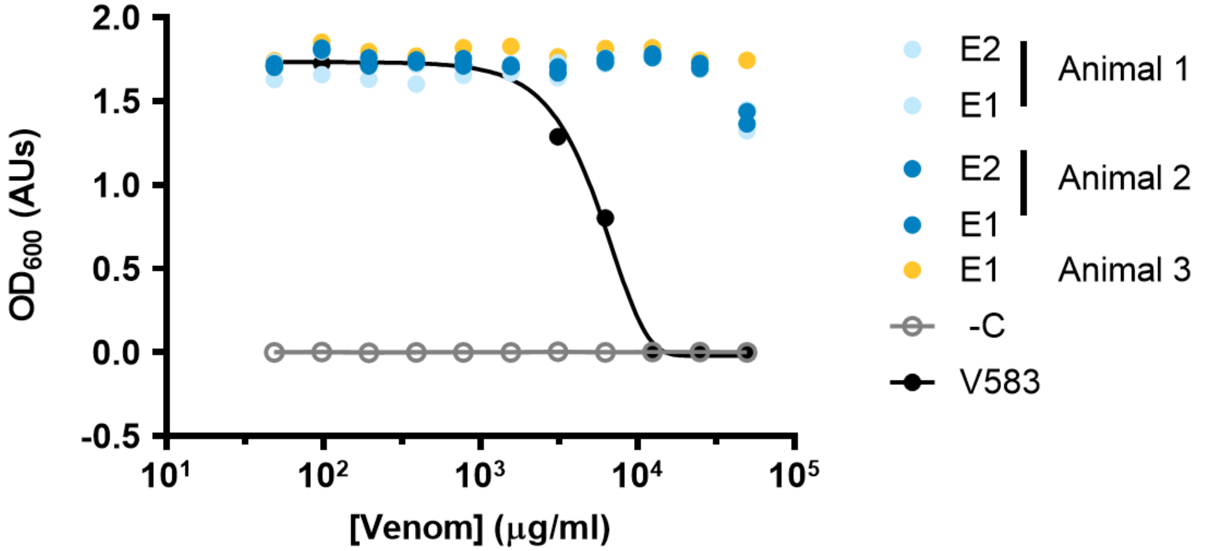

Figure S8: ***N. nigricollis*** **venom-derived** ***E. faecalis*** **isolates resist the inhibitory effects of** ***N. nigricolis*** **lyophilised venom.** The growth inhibitory effect of pooled, filter-sterilised, lyophilised *N. nigricollis* venom dissolved in brain heart infusion broth across 2-fold serial dilutions of 50 mg/ml was assessed for five *E. faecalis* isolates derived from *N. nigricollis* venom and the reference isolate V583 after 24 hr shaken incubation at 37°C by turbidity assessment at 600 nm. Data representative of 3 independent replicate experiments.

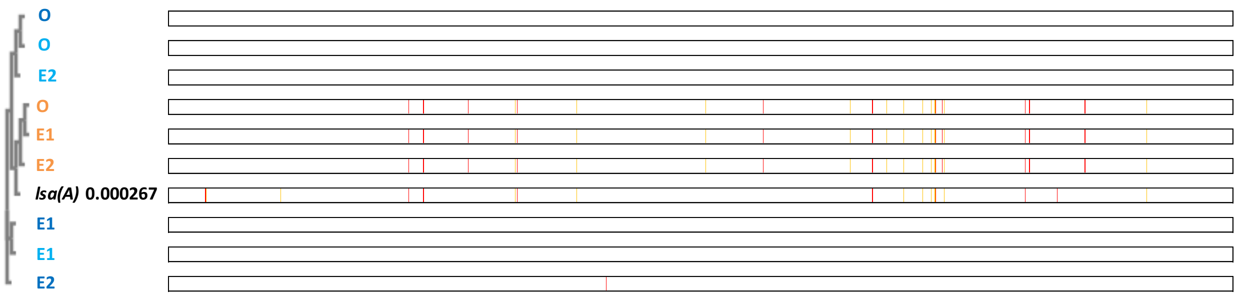

Figure S9: Multiple sequence alignment of the *lsa(A)* gene reinforces the clustering of the *N. nigricollis*-derived *E. faecalis* isolates. Blinded MSA of the *lsa(A)* antibiotic resistance gene sequence across the nine *E. faecalis isolates* obtained from *N. nigricollis* oral swabs (O) and two consecutive envenomation samples (E1 and E2) from three independent animals (light blue (N.nig01), dark blue (N.nig02) and orange (N.nig03)) defines two alleles distinct to the TX0263 reference strain gene sequence (accession no. AY737526.1). Base conservation is defined by similarity to the animal 1 and 2 *lsa(A)* sequence using BoxShade v.3.3.1 on mobyle.pasteur.fr. Each pixel column represents a different nucleotide with orange and red columns indicating increasingly different nucleotides.

Table S1: Microbial colony recovery from individual envenomation samples and apparatus collected from snakes, spiders and scorpions as observed in three types of agar.

		Agar type		
**Species**	**Sample type**	**Blood**	**MacConkey**	**Mannitol Salt**
N/A	Plating Control	ND	ND	ND
	Blinded water control	ND	ND	ND
B. arietans	oral	*	ND	ND
	envenomation 1	ND	ND	ND
	lyophilised	ND	ND	ND
	wild	ND	ND	ND
		ND	ND	ND
		ND	ND	ND
		ND	ND	ND
		ND	ND	ND
		ND	ND	ND
		ND	ND	ND
		ND	ND	ND
N. nigricollis	oral	**/F	*	ND
		**/F	*	ND
		**	ND	ND
	envenomation 1	ND	ND	ND
		***	***	ND
		**/***	***	ND
		**/***	***	ND
	envenomation 2	*/***	***	ND
		*/***	***	ND
		***	***	ND
B. atrox	oral	*/*	**	*/*
		/	*	*
	envenomation 1	*	ND	ND
		ND	ND	ND
		ND	ND	ND
C. atrox	oral	*	*	*
		*	ND	ND
	envenomation 1	ND	ND	ND
		ND	ND	ND

Table S1 continues in page 14

Table S1 continued from page 14

O. scutallatus	oral	**	ND	ND
		*	*	ND
	envenomation 1	ND	ND	ND
		ND	ND	ND
P. regalis	fang	*	*	ND
		*	ND	*
		*/***	*	ND
	envenomation 1	***	***	ND
		***/**	*	ND
		***	**	ND
	envenomation 2	***	**	ND
		*/**	ND	ND
		**	ND	ND
L. parahybana	fang	**/**	*	ND
		**/***	**	**
		**	*	**
	envenomation 1	ND	ND	ND
		ND	ND	ND
		***	***	*
		*	ND	ND
		*	ND	ND
	envenomation 2	ND	ND	ND
		*	ND	*
		ND	ND	ND
		ND	ND	ND
		**	*	ND

ND: none detected
*: 1-10 bacterial colonies
**: 11-50 bacterial colonies
***:>50 bacterial colonies
/: more than one colony type
F: fungal colony

Table S2: Isolate identification using BioMerieux biochemical strips.

Animal no.	Sample type	Species (% Confidence Interval) 1	2	3
1	O	*S. haemolyticus (47.9)*	*S. aureus (17.9)*	*S. saprophyticus (15.2)*
	E1	*S. haemolyticus (35.8)*	*S. warneri (32.0)*	*S. homini (16.1)*
	E2	S. haemolyticus (35.8)	*S. warneri (32.0)*	*S. homini (16.1)*
2	O	*S. haemolyticus (47.9)*	S. aureus (17.9)	*S. saprophyticus (15.2)*
	E1	*P. vulgaris (62.7)*	*P. penneri (35.2)*	*P. mirabilis (2.0)*
	E1	*S. haemolyticus (35.8)*	*S. warneri (32.0)*	*S. homini (16.1)*
	E2	*S. haemolyticus (36.2)*	*S. saprophyticus (19.5)*	*S. aureus (18.2)*
3	O	*S. cohnii ssp cohnii (35.5)*	*S. warnerii (19.7)*	*S. capitis (15.5)*
	E1	*S. haemolyticus (35.8)*	*S. saprophyticus (32.0)*	*S. homini (16.1)*
	E2	*S. haemolyticus (36.2)*	*S. saprophyticus (19.5)*	*S. aureus (18.2)*

O: oral swab sample
E1: envenomation 1
E2: envenomation 2

Table S3: Comparative genomic analysis of the venom-derived *E. faecalis* isolates.

		Genome assembly metrics					PlasmidFinder v.1.3				*repA-2* BLASTn	
**Animal no.**	**Isolate origin**	**Size (Mb)**	**GC mol%**	**No. of contigs**	**Contig N50 (bp)**	**Average coverage**	**Plasmid**	**Gene**	**Coverage (%)**	**Identity (%)**	**Identity (%)**	**Coverage (%)**
1	O	3.24	36.8	111	118,306	85	pTEF2	*repA-2*	99.40	96.22	96.15	100
	E1	3.24	36.8	112	153,505	210	pTEF2	*repA-2*	99.41	96.22	96.15	100
	E2	3.04	37.1	150	44,899	44	pTEF2	*repA-2*	91.27	96.22	96.25	100
2	O	3.24	36.8	109	152,238	207	pTEF2	*repA-2*	-	-	97.14	99
	E1	3.24	36.8	110	92,973	92	pTEF2	*repA-2*	91.27	96.22	96.25	100
	E2	3.24	36.8	113	137,718	72	pTEF2	*repA-2*	63.00	98.90	98.90	63*
											93.63	47*
3	O	2.9	37.2	28	347,828	315	-	-	-	-	90.45	39
	E1	2.91	37.2	30	262,705	297	-	-	-	-	90.45	39
	E2	2.91	37.2	47	100,319	61	-	-	-	-	90.45	39

O: oral sample

E1: envenomation 1

E2: envenomation 2

*: gene split across two contigs resulting in only the larger portion (63% coverage) being identified by PlasmidFinder v1.3, whereas both halves were identified by BLASTn (63% coverage on one contig, 47% coverage on another)

Table S4: Comparison of pTEF plasmid genomic elements in venom-resistant *E. faecalis* genomes groups isolates by animal of origin.

		Average base reads per plasmid			Average base read ratio		
**Animal no.**	**Isolate origin**	**pTEF1**	**pTEF2**	**pTEF3**	**pTEF1:pTEF2**	**pTEF2:pTEF3**	**pTEF1:pTEF3**
1	O	5.245	6.524	4.031	0.8	1.62	1.3
	E1	16.007	19.556	12.68	0.82	1.54	1.26
	E2	9.726	9.086	5.896	1.07	1.54	1.65
2	O	15.196	17.444	10.808	0.87	1.61	1.41
	E1	5.081	6.617	4.29	0.77	1.54	1.18
	E2	4.407	5.483	3.504	0.8	1.56	1.26
3	O	1.472	5.738	8.639	0.26	0.66	0.17
	E1	1.643	6.179	8.908	0.27	0.69	0.18
	E2	0.388	1.604	2.321	0.24	0.69	0.17

O: oral sample

E1: envenomation 1

E2: envenomation 2

Table S8: List of resistance genes present in each *E. faecalis* isolate for select antimicrobial classes.

		Antimicrobial class					
**Animal no.**	**Isolate origin**	**Aminoglycosides**	**Glycolipids**	**Macrolides**	**Streptogramins**	**Phenicols**	**Tetracyclines**
1	O	None	None	None	*lsaA*	None	None
	E1	None	None	None	*lsaA*	None	None
	E2	None	None	None	*lsaA*	None	None
2	O	None	None	None	*lsaA*	None	None
	E1	None	None	None	*lsaA*	None	None
	E2	None	None	None	*lsaA*	None	None
3	O	None	None	None	*lsaA*	None	None
	E1	None	None	None	*lsaA*	None	None
	E2	None	None	None	*lsaA*	None	None

O: oral swab sample

E1: envenomation 1

E2: envenomation 2
